# Photoelectrochemical Hydrogen Production System Using Li-Conductive Ceramic Membrane

**DOI:** 10.3390/membranes12121189

**Published:** 2022-11-25

**Authors:** Ihor A. Rusetskyi, Leonid L. Kovalenko, Michail O. Danilov, Ivan A. Slobodyanyuk, Sergii S. Fomanyuk, Vitaliy O. Smilyk, Anatolii G. Belous, Gennadii Ya. Kolbasov

**Affiliations:** V.I. Vernadskii Institute of General and Inorganic Chemistry of the Ukrainian NAS, 32/34 Academic Palladin Avenue, 03142 Kyiv, Ukraine

**Keywords:** photoelectrochemical cell, hydrogen evolution, lithium-lanthanum titanate, ceramic Li conducting membrane, photocurrent

## Abstract

Based on the LiLaTiO_3_ compound, a ceramic membrane for a photoelectrochemical cell was created. The microstructure, phase composition, and conductivity of a semiconductor photoelectrode and a ceramic membrane were studied by using various experimental methods of analysis. A ceramic Li conducting membrane that consisted of Li_0.56_La_0.33_TiO_3_ was investigated in solutions with different pH values. The fundamental possibility of creating a photoelectrochemical cell while using this membrane was shown. It was found that the lithium-conductive membrane effectively works in the photoelectrochemical system for hydrogen evolution and showed a good separating ability. When using a ceramic membrane, the pH in the cathode and anode chambers of the cell was stable during 3 months of testing. The complex impedance method was used to study the conductive ceramic membrane in a cell with separated cathode and anode chambers at different pH values of the electrolyte. The ceramic membrane shows promise for use in photoelectrochemical systems, provided that its resistivity is reduced (due to an increase in area and a decrease in thickness).

## 1. Introduction

One of the most promising and environmentally friendly methods for hydrogen evolution is the method of photoelectrocatalytic decomposition of water under the influence of sunlight while using semiconductor materials. For this, a photoelectrochemical (PEC) cell can be used, which consists of a semiconductor photoanode [1], based on A^ІІ^В^VI^ semiconductors, and a cathode immersed in an electrolyte solution. А^ІІ^В^VI^ semiconductors, in particular CdSе, are characterized by a relatively small band gap (E_g_ = 1.7 eV) and intense direct transitions, which allow for the efficient absorption of visible light in a relatively thin (several hundred nm) photoactive layer [2]. This semiconductor is stable in a sulfide solution [3,4,5,6], and the spectral dependence of the normalized value of the short-circuit current (I_sc_) has a limit for CdSe at λ ≥ 750 nm.

When narrow-gap semiconductors are used in photoelectrochemical cells, the magnitude of the photopotential is insufficient to decompose water, or the process is running at a low efficiency. To solve this problem, one can replace the anodic oxygen-evolution reaction with another, such as, for example, the reaction of oxidation sulfide ions 2S^2-^ + 2p^+^ → S_2_^2-^ [7,8,9,10,11]. The polysulfide system is also a protector, since the photooxidation of the semiconductor has not been practically observed in it due to the high rate of oxidation of sulfide ions [8,9]. This process takes place with less overvoltage in the electrochemical system, which allows one to more efficiently convert solar energy. In addition, for a more efficient production of hydrogen, it is necessary for the cathode chamber to be filled with electrolyte with pH = 1 – 2 and the anode chamber with electrolyte with pH = 13 – 14. The difference between the pH of the solutions in the anode chamber and the cathode chamber creates a potential that facilitates the processes of water decomposition during hydrogen evolution in a photoelectrochemical cell. Based on the Nernst equation [12] and considering the H^+^ transfer coefficient [13], the difference will be ~ 0.158 V. In this case, the total reaction occurring at the cathode in an acidic solution will proceed according to the mechanism 2H^+^ + 2e^−^ → H_2_. For the long-term operation of such systems, it is necessary to stabilize the composition of the anolyte and catholyte (as well as pH), which can be done by using a membrane, which excludes changes in pH and in the concentration of ions that are involved in an electrochemical reaction. Such membranes must have a high selectivity, a good electrical conductivity, a high mechanical strength and chemical resistance, and a long service life.

In recent years, new solid-state ion-conducting materials (conductors of the second class) with a high ionic conductivity, so-called superionic conductors (SCs) or solid electrolytes (fast ion conductors), have received increasing attention. As is known, under normal conditions, ion transfer in ordinary solids—both crystalline and amorphous ones—is not very significant, and at room temperature, their conductivity σ does not exceed 10^−10^–10^−12^ Ohm^−1^ cm^−1^ [14]. Currently, a large number of solid electrolytes are known to exist in which conductivity is provided by a variety of cations (single-, double- and triple-charged cations (Аg^+^, Cu^+^, Li^+^, Na^+^, К^+^, Rb^+^, Тl^+^, Cs^+^, Cа^2+^, Zn^2+^, Mg^2+^, Рb^2+^, Аl^3+^, Sс^3+^, Cе^3+^, Еu^3+^)) as well as by anions (F^−^, Cl^−^, Вr^−^, О^2−^, S^2−^). Ionic conductivity of polycrystalline inorganic ion-conducting compounds was carried out due to structural features. The structure contains a rigid crystalline framework of tetragonal channels, where ion transport takes place, as well as a sufficient number of vacancies that provide the possibility of free migration of ions. Most ionic conductors have a high-temperature conductivity. Low-temperature ionic conductivity has a limited number of compounds. At present, the best inorganic polycrystalline lithium-ion solid electrolytes with a defective perovskite structure (ABO_3_) are lanthanum-lithium titanates (LiLaTiО_3_) Li_3*x*_La_(2/3)-_*_х_*□_(1/3)-2*x*_TiO_3_ (0 < *x* < 0.16) (□—lithium conductivity of the material by vacancies) [15], which exhibit a high ionic conductivity at room temperature (at *х* = 0.11, the conductivity is σ_20 °C_ = 2·10^−3^ Ohm cm^−1^) [16]. The high ionic conductivity was explained by the imperfection of the structure of the tetragonal LiLaTiO_3_; namely, in the A sublattice, the Li and La ions are only partially populated by a certain amount, which leads to the formation of vacancies of a certain concentration, which contributes to the movement of lithium ions along vacancies and through square, flat bottlenecks between the planes formed by four adjacent oxygen octahedra [17]. In other words, to achieve a high conductivity, lithium-ion solid electrolytes with a defective perovskite structure require not only a high concentration of conduction ions (Li^+^) but also a certain concentration of vacancies (the conduction mechanism has a percolation limitation [18]), which makes these materials promising for use in various electrochemical systems. Thus, [19] showed the possibility of creating an ion-selective (Li^+^) electrode based on LiLaTiO_3_ ceramics for detecting Li^+^ ions in anhydrous solutions. This is done through the mechanism of Li^+^ ion exchange at the ceramic/solution interface.

**Figure 1:**
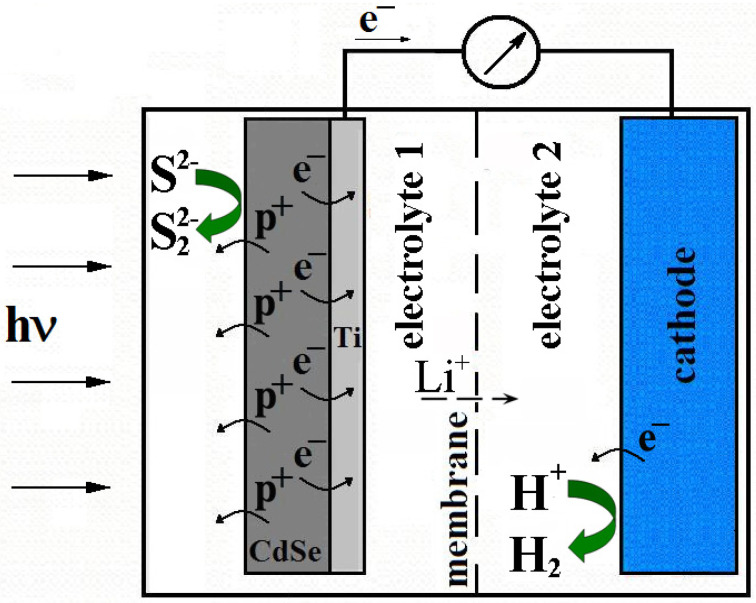
Scheme of a photoelectrochemical cell with a ceramic membrane.

We proposed using a Li_0.56_La_0.33_TiО_3_ (LiLaTiО_3_) ceramic membrane in a photoelectrochemical system [20] (Figure 1) to separate the cathode and anode chambers, which has a high conductivity with respect to lithium ions at room temperature (20 °C) [16]. This membrane has a high chemical resistance in various environments, which makes it possible to use it for a long time.

To ensure the conductivity of the system with lithium ions’ ratio and to minimize the influence of the concentration gradient, lithium ions were added to the electrolyte. The composition of the system under study, as a photoanode, included CdSe—an electrode with an electronic type of conductivity. To increase the photocorrosion resistance, the semiconductor electrode was placed in a polysulfide solution. Pt was used as a cathode, which has a low hydrogen-evolution overvoltage, and was immersed in a solution of 30% H_2_SO_4_ + 1M Li_2_SO_4_.

The aim of our work was to determine the possibility of using a ceramic membrane based on LiLaTiO_3_ in a photoelectrochemical cell for hydrogen evolution.

## 2. Experimental Section

Lithium-lanthanum titanate LiLaTiO_3_ was obtained by solid-phase reactions. The highly pure starting reagents were: La_2_O_3_, Li_2_CO_3_, TiO_2_ rutile. The homogenizing grinding of stoichiometric amounts of the starting reagents, as well as the synthesized mixture, was carried out in a planetary centrifugal mill Retsch PM-100 (Kiwa International Cert GmbH, Hamburg, Germany). The fusion of the mixture was carried out at 1050 °C for 2 h. The resulting blanks were pressed and sintered in an air atmosphere at 1300 °C for 2 h. From the obtained blanks, discs with a thickness of 0.7 mm were cut, 1.35 mm and 20.5 mm in diameter. The design of the PEC cell allows for tightly fixed membranes and prevents the electrolytes from mixing. The density of ceramic LiLaTiO_3_ samples was measured by the pycnometric and geometric methods. The microstructure of the ceramic was examined by using a JEOL JSM-6510 (JEOL Ltd., Tokyo, Japan) microscope. To study the conductivity of the LiLaTiO_3_ ceramic, the contacts were prepared by firing them on a silver-containing paste. For impedance studies in the 32 MHz–0.1 Hz range, an impedance analyzer 1260A Impedance/Gain-Phase Analyzer (Solartron Analytical, Farnborough, UK) was used. The electrical equivalent circuit and the values of its components were determined by using the ZView computer program. The temperature-dependence conductivity of the ceramic sample from Li_0.56_La_0.33_TiO_3_ was determined according to the procedure described in [21]. X-ray diffraction (XRD) analysis was performed on a DRON-4-07 X-ray diffractometer.

The current-voltage characteristics were measured on a model of a photoelectrochemical cell with two different electrodes with an area of 2 cm^2^ (CdSe electrode) and 1 cm^2^ (Pt electrode) by using a P-8S potentiostat (Elins, Zelenograd, Russia). The design of the cell made it possible to separate the cathode and anode chambers with a membrane (Figure 1). The following solutions served as electrolytes (anode/cathode chambers, respectively): 1 M NaOH + 1 M Na_2_S + 10% LiOH/30% H_2_SO_4_ + 1 M Li_2_SO_4_ and 1 M NaOH + 1 M Na_2_S + 10% LiOH/30% KOH + 2 M LiOH. To determine the load characteristics, a polysulfide solution of 1 M NaOH + 1 M Na_2_S was used, similarly to [5,9,22,23]. Comparative current-voltage characteristics were measured in a cell using electrolytes of 1M Na_2_S + 1M NaOH и 1M NaOH + 1M Na_2_S + 10% LiOH for the CdSe electrode, as well as 30% KOH and 30% KOH + 2M LiOH for the Pt electrode. A silver chloride electrode connected through a salt bridge was used as a reference electrode. A KGM 9-70 (Yugra Invest, TOO; Kazakhstan, Pavlodar halogen lamp was used as a light source (lamp power was 70 W). The incident light power was measured using a PD300-UV (Ophir-Spiricon, North Logan, UT, USA) head photodiode and a NOVA II display (Ophir-Spiricon, North Logan, UT, USA).

The photosensitive CdSe semiconductor film was formed by the electrochemical method on a 0.4-mm-thick VT1-0 titanium substrate. The substrate was preliminarily degreased in acetone, followed by etching for 1–2 min in a mixture of acids: HF, 0.75 mol L^−1^; HNO_3_, 3.17 mol L^−1^. Then, electrochemical treatment was carried out in a solution of 0.7 mol L^−1^ H_2_SO_4_ (mode: E = 0.2–0.65 V; sweep 10 mV s^−1^; 5 cycles). The electrochemical deposition of a semiconductor CdSe film was carried out in the potentiostatic mode on a titanium substrate at the potential E = −0.6 V (±0.03 V) relative to a silver-chloride reference electrode. The current density was j = 0.7–2 mA cm^−2^, and the time was 30 min. For electrolysis, a sulfuric acid electrolyte was used with the following components: H_2_SO_4_, 0.7–2 mol L^−1^; H_2_SeO_3_, 0.003–0.005 mol L^−1^; CdSO_4_, 0.02–0.03 mol L^−1^. The annealing of the CdSe electrode was carried out in an air atmosphere at 470 °C for 3 h. Then, the surface was activated in an aqueous solution: HCl, 5 mol L^−1^, HNO_3_, 0.27 mol L^−1^ for 4–5 s at room temperature. The surface of the CdSe semiconductor film was studied using a scanning electron microscope JSM 6700F (JEOL Ltd., Tokyo, Japan).

## 3. Results and Discussion

**Figure 2:**
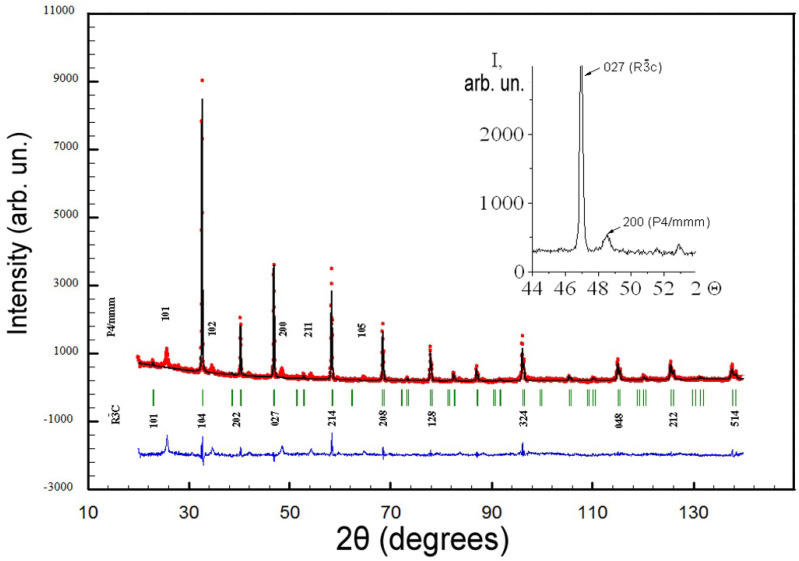
X-ray diffraction pattern of LaLiTiO_3_ ceramic sample, which contains two phases with different symmetries: R$\bar{3}$c and P4/mmm.

The results of the XRD analysis (Figure 2) of a sintered ceramic sample with the composition LiLaTiO_3_ show that the main phase is solid solutions with a perovskite structure of the rhombohedral system (space group R$\bar{3}$c) with the unit cell parameters: a ≈ 5.48 Å and c ≈ 13.42 Å; similar results were obtained in [21,24,25].

In addition, the presence of low-intensity peaks of the P4/mmm tetragonal phase was observed, corresponding to the same chemical composition of LiLaTiO_3_ as in the rhombohedral phase.

According to [26], a tetragonal phase in the form of locally ordered nanoregions was even observed in hardened samples. Table 1 shows the structural parameters of a LiLaTiO_3_ ceramic sample. 

**Figure 3:**
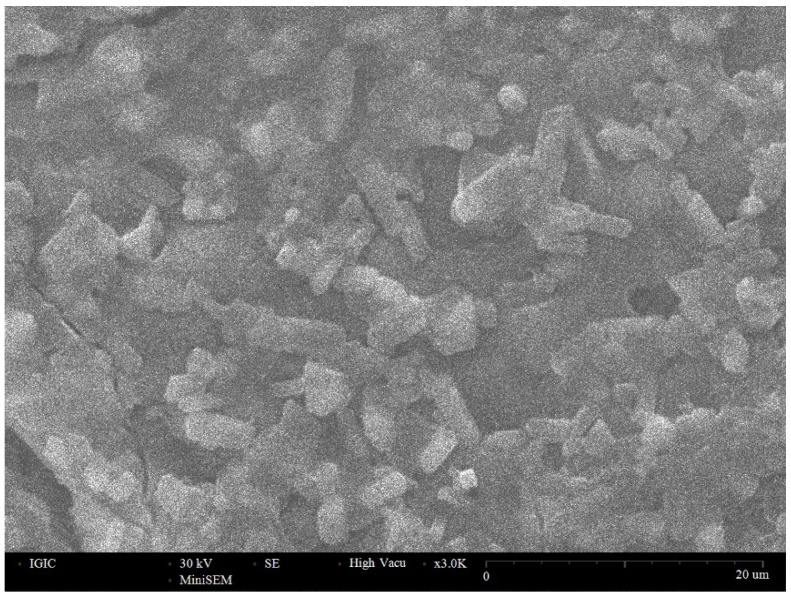
SEM micrograph of a cleavage of a LiLaTiO_3_ ceramic sample.

Figure 3 shows a micrograph of a cleavage of a LiLaTiO_3_ ceramic sample. As can be seen from the photograph, the grain size of the ceramic is 2–10 microns.

When measuring the density of LiLaTiO_3_ ceramic samples by the geometric method, the closed porosity contributes to the obtained value. Therefore, a comparison of values obtained by the pycnometric and geometric methods makes it possible to estimate the value of the closed porosity. For the samples under investigation, the value of geometric density was close to the values of pycnometric density and differed by up to 10–15%.

**Figure 4:**
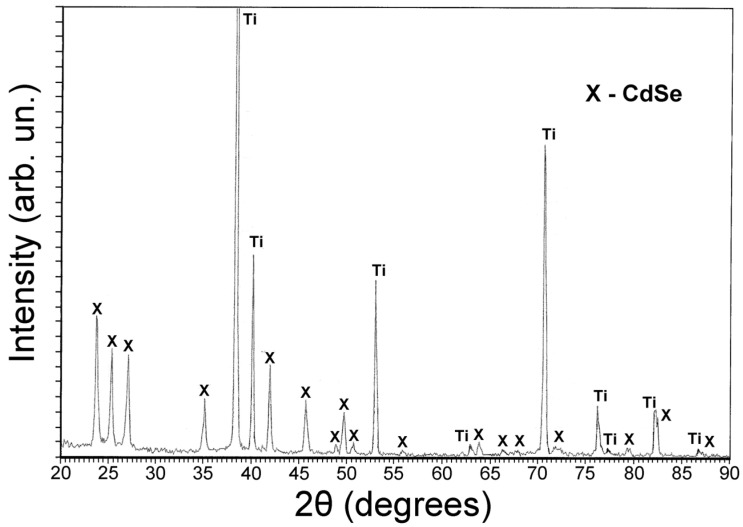
X-ray diffraction pattern of the CdSe electrode surface.

The XRD analysis of the surface of a CdSe electrode obtained by electrochemical deposition on a titanium substrate and subsequent annealing showed the formation of a hexagonal CdSe phase (Figure 4).

**Figure 5:**
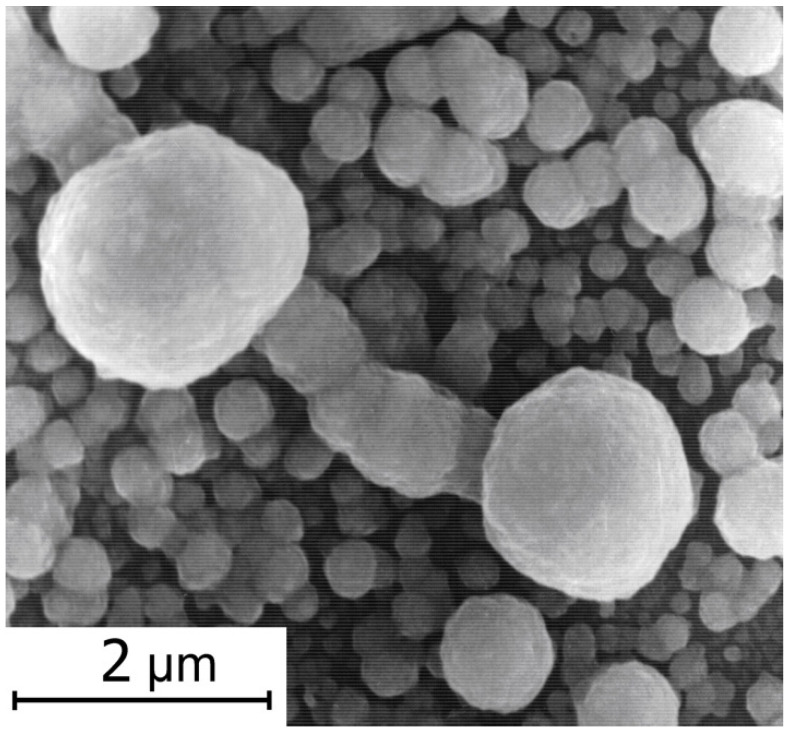
SEM micrograph of the CdSe electrode surface obtained by electrochemical treatment.

It was found from SEM microscopic studies that the average size of electrochemically deposited CdSe particles is less than 2 μm (Figure 5).

**Figure 6:**
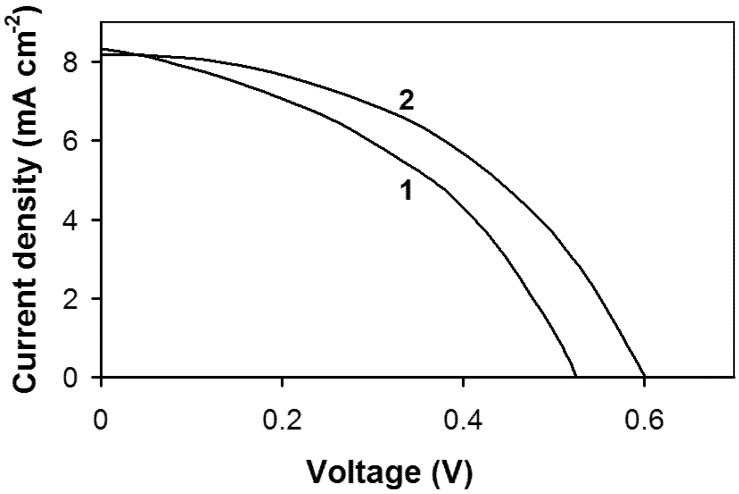
Loading characteristics of a CdSe photoelectrode after annealing (1) and after its activation (2). The solution was: 1M Na_2_S + 1M NaOH. The counter electrode is platinum. Lighting power is 16 mW cm^−2^.

Figure 6 shows the load characteristics of the CdSe-Pt cell in the polysulfide system after annealing of the CdSe photoelectrode and its activation. Figure 6 shows that the surface activation leads to an increase in the efficiency of the photoelectrode. Characteristically, after activation of the photoelectrode, the open-circuit voltage increases most significantly, while the short-circuit current changes little.

The current–voltage characteristics of the cell were measured in solutions with different pH values and a Li_0.56_La_0.33_TiO_3_ ceramic membrane (thickness 0.7 mm), where platinum plates (with an area of 1 cm^2^) served as electrodes. It was found that the cathode currents reach limiting values of 300–450 μA, after which they do not increase.

**Figure 7:**
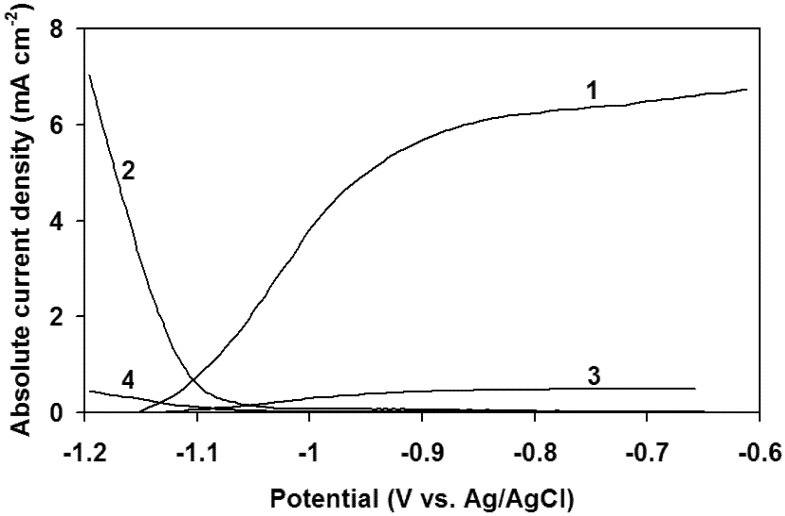
Comparative current–voltage characteristics of various electrodes with CdSe photoanode (1, 3) (light power 16 mW cm^−2^) and Pt electrode (2, 4) in electrolytes: 1 M Na_2_S + 1 M NaOH (1); 30% KOH (2); 1 M NaOH + 1 M Na_2_S + 10% LiOH (3); 30% KOH + 2 M LiOH (4). Curves (3, 4) obtained in the cell with electrode chambers separated by a Li_0.56_La_0.33_TiO_3_ ceramic membrane.

Figure 7 shows the comparative current–voltage characteristics of the CdSe photoanode and the platinum cathode in the cell without a membrane (curves 1, 2) and with electrode chambers separated by a Li_0,56_La_0,33_TiO_3_ ceramic membrane (thickness 0.7 mm) (curves 3, 4).

As can be seen from Figure 7, the photoanode ensures a hydrogen evolution on platinum in both cases. This is evidenced by the intersection of curves 1 and 2, as well as curves 3 and 4. For the cell with a ceramic membrane, the currents flowing in the system are lower than in a cell without a membrane. This indicates a high internal resistance of the membrane.

**Figure 8:**
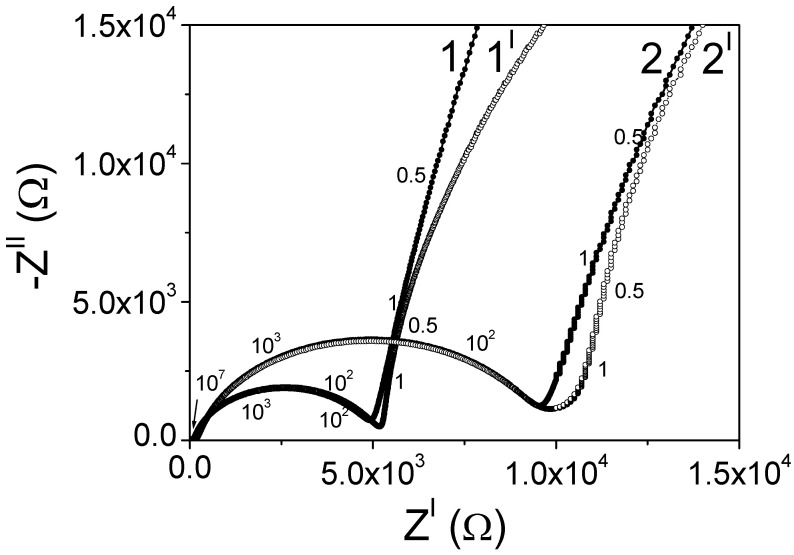
Impedance diagrams in Nyquist coordinates of an electrochemical cell in solutions of 30% KOH + 2M LiOH (1^I^, 2^I^) and 30% H_2_SO_4_ + 1M Li_2_SO_4_ (1, 2), where ceramic plates of LiLaTiO_3_ were used as a membrane with thicknesses (mm) of: 0.7 (1,1^I^); 1.35 (2,2^I^). The numbers on the curves are the frequency in Hz.

The complex impedance method was used to study the conductivity of a cell with separated cathode and anode chambers at different pH values and platinum electrodes with an area of 1 cm^2^, where the membrane was a LiLaTiO_3_ ceramic material with different thicknesses (0.7 mm and 1.35 mm). Figure 8 shows the impedance hodographs in alkaline and acidic solutions.

When compiling an equivalent electrical circuit of the studying system, it is necessary to take into account the effect of the charge transfer process at the membrane–electrolyte interface, charge transfer in the membrane volume, intergrain contact resistance, etc. [27,28,29]. The optimal equivalent electrical circuit of the system and the values of its parameters, obtained by using the ZView computer program, are presented in Table 2. According to the above diagram, CPE-2 and R3 correspond to the process of charge transfer at the interface; CPE-1 and R2 reflect the diffusion of lithium ions in the membrane volume, which determines membrane impedance at a low frequency (Figure 8); R1 is the intergranular contact resistance, including a small electrolyte resistance. In this case, when the thickness of the LiLaTiO_3_ membrane changed from 1.35 mm to 0.7 mm, the values of R1 and R2 were directly proportional to its thickness.

**Figure 9:**
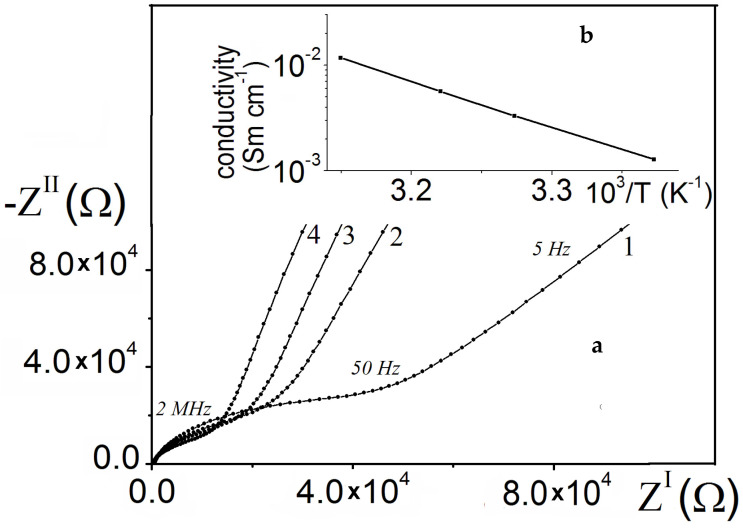
(**a**) Nyquist plots of the LiLaTiO_3_ ceramic at temperatures of, °C: (*1*) 23, (*2*) 32, (*3*) 37, (*4*) 45. (**b**) Temperature dependence of conductivity of the LiLaTiO_3_ ceramic.

Based on complex impedance plots in Nyquist coordinates obtained at different temperatures, a plot of the conductivity versus return temperature 1/Т for a Li_0.56_La_0.33_TiO_3_ ceramic sample has been constructed in a frequency area where the frequency dependence of the impedance is close to linear (Figure 9). The conductivity of the Li_0.56_La_0.33_TiO_3_ ceramic material corresponds to the values described in the literature [30].

The activation energy was calculated to be 0.27 eV, which indicates the main contribution to the bulk resistance low-frequency impedance, which was determined by the diffusion of lithium ions in the bulk of the material.

Similar results were obtained by the authors of [31]. It has been established that the thinner the thickness of the ceramic electrolyte, the lower the internal resistance and the higher the operating voltage of the lithium-air cell. Thus, with a ceramic electrolyte thickness of 0.7 mm, the operating voltage of the element is 0.46 V, with a thickness of 0.41 mm it is 1.06 V, and with a thickness of 0.25 mm it is 1.63 V (in all cases, the discharge current density was 0.4 mA cm^−2^).

**Figure 10:**
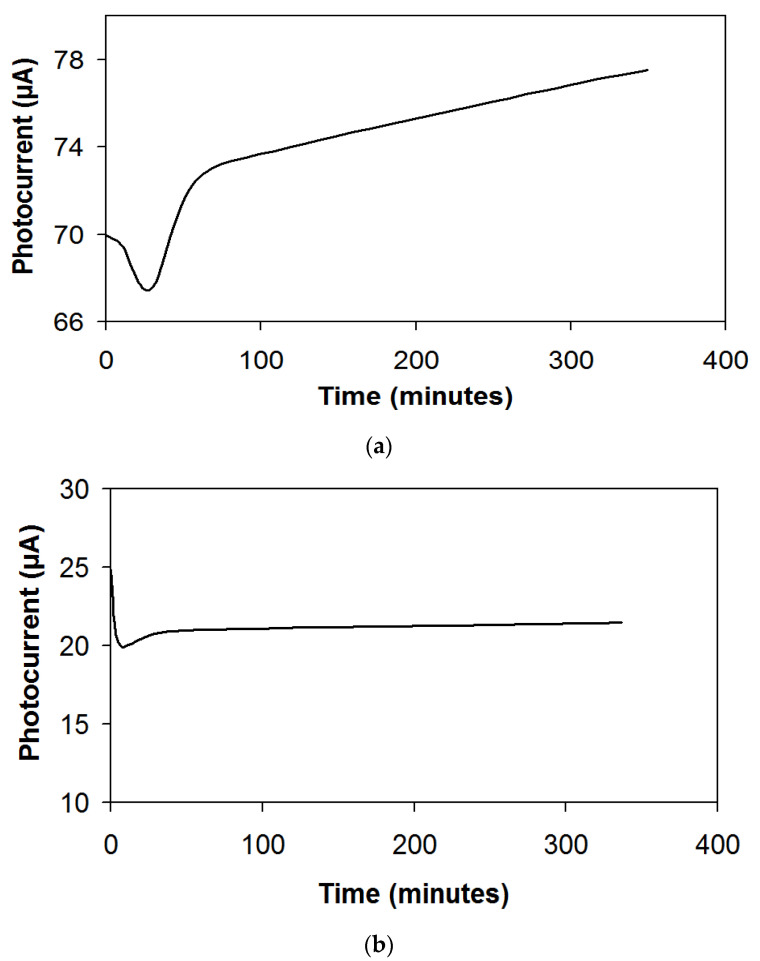
Dependence of the photocurrent on time for a PEC cell with a Li_0.56_La_0.33_TiO_3_ membrane (**a**,**b**). The electrolytes in anode/cathode chamber, respectively: (**a**) 1 M NaOH + 1 M Na_2_S + 10% LiOH/30% H_2_SO_4_ + 1 M Li_2_SO_4_; (**b**) 1 M NaOH + 1 M Na_2_S + 10% LiOH/30% KOH + 2 M LiOH. The light power was 16 mW cm^−2^.

When studying the parameters of a photoelectrochemical cell, it was found that the Li_0.56_La_0.33_TiO_3_ ceramic membranes effectively operated in the PEC system for the hydrogen evolution. A CdSe photoanode and a Pt cathode were used for measurements of the photocurrent in the photoelectrochemical cell. Figure 10 shows plots of the short-circuit photocurrent versus time for a PEC cell when the photoanode is illuminated in different electrolytes and for a LiLaTiO_3_ membrane.

In practical applications in a PEC cell, large values of the hydrogen-evolution current are realized when using alkaline and acidic electrolytes in the anode and cathode chambers, respectively (Figure 10a). Smaller values of the hydrogen-evolution current are realized when using alkaline electrolytes in the anode and cathode chambers (Figure 10b). When testing a photoelectrochemical cell under illumination with a ceramic membrane that separates the cathode and anode chambers, the pH in the cathode chamber remained stable for an operation time of 3 months.

Thus, ceramic membranes show promise for use in photoelectrochemical systems in cases where their resistivity is reduced (due to an increase in area and a decrease in thickness).

## 4. Conclusions

We showed the fundamental possibility of creating a photoelectrochemical cell for hydrogen production by using a ceramic membrane based on Li_0.56_La_0.33_TiO_3_. The parameters of a photoelectrochemical cell in alkaline and acidic electrolytes have been studied. The ceramic membrane in the electrochemical cell showed a good separating ability, but due to a high resistance, it had large polarization losses. The complex impedance method was used to study the conductivity of a membrane in a cell with separated cathode and anode chambers at various pH values. The conductivity was studied by using a membrane with a thickness of 0.7 mm to 1.35 mm. The optimal equivalent circuit and the values of its parameters were determined. It was found that when the membrane thickness changed from 1.35 mm to 0.7 mm, its resistance was directly proportional to its thickness.

## Figures and Tables

**Table 1:** Structural parameters of a LaLiTiO_3_ sample.

	R3c Phase	P4/mmm
Unit cell parameters		
a, A	5.4836(4)	5.4720(3)
c, A	13.44(1)	7.7735(6)
V, A^3^	349.9(3)	232.76(3)
Coordinates of ions *		
Ti z/c	0	0.251(4)
O1 x/a	0.53(3)	½
O3/O4 z/c	-	0.21(2)
Reliability factor		
X^2^	9.53	9.53
R_B_, %	6.74	13.5
R_f_, %	8.86	10.5
R_exp_,%	9.17	9.17

* Position of atoms for the R$\bar{3}$c phase: La (6a) 0 0 1/4; Li (18d) ½ 0 0; Ti (6b) 0 0 0; O (18e) *x* 0 ј; for the P4/mmm phase: La (1a) 0 0 0; Ti (2t) ½ ½ *z*; O1 (1f) ½ ½ 0; O2 (1h) ½ ½ ½; O3 (2s) ½ 0 z; O4 (2r) 0 ½ *z*.

**Table 2:** Equivalent circuit of the impedance and the value of its components depending on the membrane thickness and pH of the electrolyte.

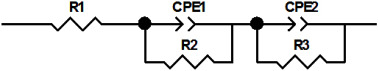			
Membrane Thickness 0.70 mm		Membrane Thickness 1.35 mm	
electrolyte 30% KOH + 2 M LiOH			
R1 (Ohm)	82.32	R1 (Ohm)	164.2
CPE1	3.895 × 10^−7^	CPE1	2.579 × 10^−7^
R2 (Ohm)	5069	R2 (Ohm)	9911
CPE2	1.038 × 10^−4^	CPE2	0.8163 × 10^−4^
R3 (Ohm)	1.3573 × 10^5^	R3 (Ohm)	2.0037 × 10^5^
electrolyte 30% H_2_SO_4_ + 1 M Li_2_SO_4_			
R1 (Ohm)	82.09	R1 (Ohm)	157.3
CPE1	4.3954 × 10^−7^	CPE1	2.3086 × 10^−7^
R2 (Ohm)	4985	R2 (Ohm)	9588
CPE2	4.2605 × 10^−5^	CPE2	3.3769 × 10^−5^
R3 (Ohm)	3.7751 × 10^5^	R3 (Ohm)	2.0456 × 10^5^

## Data Availability

Not applicable.
